# Risk factors for ventilator-associated pneumonia due to multi-drug resistant organisms after cardiac surgery in adults

**DOI:** 10.1186/s12872-022-02890-5

**Published:** 2022-11-04

**Authors:** Meizhu Wang, Xi Xu, Shuo Wu, Huiying Sun, Yan Chang, Meng Li, Xiaoxiao Zhang, Xing Lv, Zhaoxu Yang, Xinyu Ti

**Affiliations:** 1 Department of Cardiovascular Surgery, Xijing Hospital, Fourth Military Medical University, No. 127, Changle West Road, Xincheng District, 710032 Xi’an, China; 2 Department of Disease Control and Prevention, Xijing Hospital, Fourth Military Medical University, 710032 Xi’an, China; 3 Department of Pulmonary and Critical Care Medicine, Xijing Hospital, Fourth Military Medical University, 710032 Xi’an, China

**Keywords:** Ventilator-associated pneumonia, Cardiac surgery, Multidrug-resistant organisms, Risk factors

## Abstract

**Background:**

Ventilator-associated pneumonia (VAP) is one of the most common intensive care unit (ICU)-acquired infections, which can cause multiple adverse events. Due to bacterial mutation and overuse of antimicrobial drugs, multidrug-resistant organisms (MDRO) has become one of the major causes of postoperative VAP infections in cardiac patients. Therefore, this study aims to explore the risk factors for VAP with MDRO following cardiac surgery in adults.

**Methods:**

The clinical data of adult VAP patients following cardiac surgery in the hospital from Jan 2017 to May 2021 were analyzed retrospectively, and the patients were divided into the MDRO VAP group and the non-MDRO VAP group. Univariable and multivariable logistic regression analyses were performed on risk factors in patients with MDRO VAP. The species and drug sensitivity of pathogens isolated from the VAP patients were also analyzed.

**Results:**

A total of 61 VAP cases were involved in this study, with 34 cases in the MDRO VAP group (55.7%) and 27 cases in the non-MDRO VAP group (44.3%). Multivariable logistic regression analysis showed that independent risk factors for MDRO VAP included preoperative creatinine clearance rate (CCR) ≥ 86.6ml, intraoperative cardiopulmonary bypass (CPB) time ≥ 151 min, postoperative acute kidney injury (AKI) and nasal feeding. Gram-negative bacilli were the main pathogens in VAP patients (n = 54, 90.0%), with the highest rate of *Acinetobacter baumannii* (n = 24, 40.0%). Additionally, patients with MDRO VAP had a significantly longer postoperative intensive care unit (ICU) duration and higher hospitalization costs than non-MDRO VAP patients, but there was no notable difference in the 28-day mortality rate between the two groups.

**Conclusion:**

Based on implementing measures to prevent VAP, clinicians should pay more attention to patients with kidney disease, longer intraoperative CPB time, and postoperative nasal feeding to avoid MDRO infections.

## Background

Ventilator-associated pneumonia (VAP) is one of the most frequent intensive care unit (ICU)-acquired infections and one of the most common types of hospital-acquired infections in cardiac patients after surgery, with a high mortality rate [1, 2]. Importantly, VAP can cause numerous adverse events, including aspiration, atelectasis, and pulmonary edema, which may increase the incidence, length of stay, and cost of care for mechanically ventilated patients [3]. Due to bacterial mutation and overuse of antimicrobial drugs, multidrug-resistant organisms (MDRO) have become a major bacterial group for VAP infections in postoperative cardiac patients [4]. A recent study indicated that bacterial infection is a significant predictor of death one year after cardiac surgery and that MDRO infection poses great challenges to clinical treatment [5]. Thus, there is an urgent need to identify risk factors for infection in MDRO VAP patients.

*Pseudomonas aeruginosa*, *Acinetobacter baumannii* (*A. baumannii*), and *Enterobacteriaceae* are the three most common drug-resistant Gram-negative bacteria. Studies have shown that VAP caused by Gram-negative [6], and multiple drug-resistant pathogens can lead to preoperative infections (e.g., endocarditis) or postoperative infections (including wound and device infections) that affect the prognosis of patients during cardiac surgery. Furthermore, previous studies suggested that postoperative VAP infection in patients after cardiac surgery is associated with a variety of factors, including preoperative congestive heart failure, intraoperative red blood cell input volume, blood glucose levels, and length of extracorporeal circulation [7]. In addition, frequent postoperative invasive procedures like aortic balloon counterpulsation and cardiac catheter placement can also result in VAP infection in patients [8, 9].

However, the risk factors for MDRO-induced VAP after cardiac surgery in adults have not been adequately reported. Therefore, in this study, we collected data on VAP infection in adults after cardiac surgery to analyze the VAP risk factors and prognosis for MDRO infection.

## Materials and methods

### Definition and diagnostic criteria

VAP is the infections in trachea/bronchi/lungs occurred in patients with tracheal intubation (or tracheotomy and insertion of the tracheal tube) after 48 h of ventilator support treatment and within 48 h of withdrawal of tracheal intubation (or withdrawal of the ventilator but retaining the tracheal tube). According to the Guidelines for the Prevention, Diagnosis, and Treatment of Ventilator-Associated Pneumonia (2013) [10], VAP is diagnosed upon the presence of new or progressive infiltrates on chest X-ray images, and accompanied by two or more of the following: (1) Fever (> 38 °C) or hypothermia (< 36 °C). (2) Peripheral blood WBC count > 10 × 10^9^/L or < 4 × 10^9^/L. (3) Purulence secretion in the trachea and bronchus. In cases of suspected VAP, patients need to leave samples for etiological examination before the empirical use of antibiotics. Generally, the secretions extracted by endotracheal aspiration (ETA) are considered positive for a total colony count of ≥ 10^5^ cfu/ml; sometimes samples taken by protected specimen brush (PSB) and bronchoalveolar lavage (BAL) are considered positive for a total colony count of ≥ 10^3^ cfu/ml and ≥ 10^4^ cfu/ml, respectively.

Acute kidney injury (AKI) refers to a sudden decline (within 48 h) in renal function (glomerular filtration function). Its diagnostic criteria are an increase in the absolute value of serum creatinine by 0.3 mg/dl (26.5 µmol/l) or a urine output < 0.5 ml/ (kg. h) for more than 6 h.

MDRO is bacteria that are not sensitive to three or more types of antibacterial drugs.

### Patient and design

This study collected patients admitted to the ICU after cardiac surgery between January 2017 and May 2021. During this period, a total of 5,919 patients undergoing thoracotomy received ventilator-assisted breathing after surgery, of whom 766 were intubated for more than 48 h, and 61 developed VAP. Then, the 61 cases were divided into two groups, with 27 in the MDRO VAP group and 34 in the non-MDRO VAP group (Table 1). The inclusion criteria were as follows: (1) Age ≥ 18 years old. (2) Mechanical ventilation > 48 h. (3) Meeting the VAP diagnostic criteria. Exclusion criteria: (1) The type of surgery was interventional or thoracoscopy. (2) Pre-existing lung infection on admission. The preoperative use of antibiotics: 0.5 h before surgery and every 3 h after the start of surgery, with cefazolin as the antimicrobial prophylaxis, up to three times. Those allergic to cefazolin were treated with the current generation of cephalosporin. This study has been approved by the ethics committee of the First Affiliated Hospital of Xi’an Air Force Military Medical University.

**Table 1:** Baseline characteristics for patients with VAP [M (QL, QU)/n (%)]

Characteristic	VAP (n = 61)	Non-MDRO VAP (n = 34)	MDRO VAP (n = 27)	***P***
**Age(years)**	56.0(46.0,63.0)	57.5(47.3,64.0)	52(45,61)	0.358
**Gender**				0.840
Male	49(80.3)	27(79.4)	22(81.5)	
Female	12(19.7)	7(20.6)	5(18.5)	
**BMI (kg/m** ^**2**^ **)**	23.4(21.9,26.5)	23.4(21.9,25.4)	24.2(21.3,28.1)	0.224
**Creatinine clearance**	74.9(55.1,91.4)	67.5(50.3,84.4)	86.6(59.4,102.2)	0.093
**Blood glucose (mmol/dL)**	5.68(4.70,6.76)	5.57(4.47,6.31)	5.72(4.89,6.98)	0.523
**Hypertension**				0.027
No	30(49.2)	21(61.8)	9(33.3)	
Yes	31(50.8)	13(38.2)	18(66.7)	
**Diabetes**				0.696
No	58(95.1)	32(94.1)	26(96.3)	
Yes	3(4.9)	2(5.9)	1(3.7)	
**History of smoking**				0.924
Never	39(63.9)	21(61.8)	18(66.7)	
Now	17(27.9)	10(29.4)	7(25.9)	
Once	5(8.2)	3(8.8)	2(7.4)	
**History of previous heart surgery**				0.463
No	47(77.0)	25(73.5)	22(81.5)	
Yes	14(23.0)	9(26.5)	5(18.5)	
**Pulmonary hypertension**				0.374
No	54(88.5)	29(85.3)	25(92.6)	
Yes	7(11.5)	5(14.7)	2(7.4)	
**Preoperative ICU days**				0.090
< 3	54(88.5)	28(82.4)	26(96.3)	
≥ 3	7(11.5)	6(17.6)	1(11.5)	
**Preoperative hospital days**				0.044
< 3	21(34.4)	8(23.5)	13(48.1)	
≥ 3	40(65.6)	26(76.5)	14(51.9)	
**Type of surgery**				0.465
**Valves**	21(34.4)	13(38.2)	8(29.6)	
**Coronary Graft**	4(6.6)	3(8.8)	1(3.7)	
**Valve + Coronary Graft**	26(42.6)	14(41.2)	12(44.4)	
**Aorta**	9(14.8)	3(8.8)	6(22.2)	
**Other** ^**a**^	1(1.6)	1(2.9)	0(0.0)	

a: Only one case was classified as other surgery, which belongs to congenital heart disease

### Data collection

Patient information was collected from the Xinglin Hospital infection system and the Jiahe electronic medical record system. The preoperative laboratory data were recorded from admission to the last examination before surgery. For the postoperative data, if the same pathogen was detected multiple times at the same site in a VAP patient, only the first data would be recorded. The collected data included age, gender, diabetes history, smoking history, previous cardiac surgery history, pulmonary artery pressure, preoperative ICU days, preoperative hospital days, and type of operation in ICU patients after cardiac surgery from January 2017 to May 2021.

### Etiological examination

The quality control strains *Staphylococcus aureus* (ATCC29213), *Escherichia coli* (ATCC25922), *Klebsiella pneumoniae* (ATCC700603), and *Pseudomonas aeruginosa* (ATCC27853) were all purchased from the Clinical Laboratory Center of the National Health Commission. Airway secretions were required to be drawn for pathogen culture and pathogenic examination at the following times: (1) Once within the first 48 h of treatment. (2) Once again before extubation of the trachea for patients treated for more than 48 h but less than 7 days. (3) At least twice a week for patients treated for more than 7 days. (4) Promptly extracting the secretions for patients with changes such as fever/respiratory symptoms/increased lung signs within 48 h after removing tracheal intubation (or withdrawing the ventilator but retaining the tracheal tube). (5) Only once when the ventilator was withdrawn for those who just kept the tracheal tube. After that, the specimens were inoculated into the corresponding media and identified using the VITEKMS microbial mass spectrometer (BioMérieux, France) according to the fourth edition of the National Clinical Laboratory Procedures. The drug susceptibility was carried out using the VITEK COMPANT 2 automatic microbial identification system to detect the MIC Value and a supplementary testing was performed via disk diffusion method (KB method). The interpretation of the results was based on the latest standard of the American Clinical and Laboratory Standards Institute (CLSI) M100.

### Data analysis

A database was established using Epidata 3.1 and data analysis was applied via the SPSS 24.0 statistical software. Normality tests were performed using histograms of normal curves. The drug susceptibility results were statistically analyzed by WHONET5.6 software. The continuous variables were expressed as median (M) and quartile (Q) for the central tendency and the dispersion trend of measurements that did not follow a normal distribution. The continuous variables were divided into 2 subgroups through receiver operating characteristic (ROC) curves according to their cutoff values identified by local control status. Comparisons of categorical variables (expressed in frequency or percentage) were performed using the Chi-square test or the Wilcoxon Rank Sum test. The impact of potential risk factors on VAP was analyzed using univariable and multivariable logistic regression analyses with non-MDRO-infected individuals as the reference group. Normally distributed variables were analyzed using the independent samples t-test and non-normally distributed variables were analyzed using the Mann-Whitney U test for comparison between the two groups. A two-tailed *P* value < 0.05 was considered statistically significant.

## Results

### Risk factors for VAP infection in patients with MDRO after cardiac surgery

In the 61 patients with VAP, there were no significant differences in baseline characteristics between the non-MDRO and MDRO groups, except for the number of preoperative hospital days (*P* = 0.044) (Table 1). In addition, we further analyzed the risk factors for VAP infection in patients with MDRO after cardiac surgery. Univariate logistic regression analysis showed that preoperative-related factors included hypertension (*P* = 0.030), creatinine clearance (*P* = 0.013), and preoperative hospital days (*P* = 0.048); intraoperative correlates included length of the aortic block (*P* = 0.019); and postoperative correlates included postoperative creatinine (*P* = 0.049) and nasogastric tubing (*P* = 0.013), all of which were statistically significant risk factors for MDRO-infected VAP patients (Table 2). Moreover, results suggested that preoperative creatinine clearance ≥ 86.6 (*P* = 0.005), intraoperative extracorporeal circulation time ≥ 151 min (*P* = 0.043), postoperative AKI (*P* = 0.024), and nasogastric tubing (*P* = 0.020) were independent risk factors for MDRO VAP infection in patients after cardiac surgery (Table 3).

**Table 2:** Univariable logistic analysis of the risk factor for the patient with MDRO VAP

Variables	OR (95%CI)	***P***
**Pre-operative related factors**		
BMI (kg/m^2^)		
< 26.7	1.000	
≥ 26.7	3.412 (0.998–11.663)	0.050
Hypertension		
No	1.000	
Yes	3.231 (1.122–9.303)	0.030
Creatinine clearance		
< 86.6	1.000	
≥ 86.6	4.154 (1.351–12.768)	0.013
Blood glucose (mmol/dL)		
< 6.51	1.000	
≥ 6.51	2.269 (0.725–7.099)	0.159
Preoperative stay in ICU (days)	1.000	
< 3		
≥ 3	0.179 (0.020–1.593)	0.123
Pre-operative hospitalization (days)		
< 3	1.000	
≥ 3	0.331 (0.111–0.990)	0.048
**Surgery-related factors**		
Duration of surgery (hours)		
< 6.6	1.000	
≥ 6.6	0.500 (0.176–1.422)	0.194
Red blood cell input (u)		
< 7.5	1.000	
≥ 7.5	0.417 (0.126–1.382)	0.152
Length of extracorporeal circulation (minutes)		
< 151	1.000	
≥ 151	3.560 (1.002–12.640)	0.050
Length of aortic block (minutes)		
< 77	1.000	
≥ 77	4.539 (1.289–15.991)	0.019
**Postoperative related factors**		
Cardiac catheterization		
No	1.000	
Yes	0.563 (0.198–1.601)	0.281
Impaired consciousness		
No	1.000	
Yes	2.078 (0.744–5.806)	0.163
Postoperative creatinine (mg/dl)		
< 2.7		
≥ 2.7	3.750 (1.007–13.965)	0.049
Postoperative creatinine clearance		
< 24.49	1.000	
≥ 24.49	0.133 (0.015–1.220)	0.074
Acute kidney injury		
No	1.000	
Yes	2.672 (0.929–7.757)	0.071
Tracheotomy		
No	1.000	
Yes	2.579 (0.882–7.539)	0.083
Secondary intubation		
No	1.000	
Yes	1.662 (0.569–4.856)	0.353
Nasogastric tubing		
No	1.000	
Yes	4.640 (1.376–15.643)	0.013

**Table 3:** Multivariable logistic analysis of the risk factor for the patient with MDRO VAP

Variables	OR (95%CI)	***P***
Creatinine clearance		
< 86.6	1.000	
≥ 86.6	7.834 (1.832–33.497)	0.005
Length of extracorporeal circulation (minutes)		
< 151	1.000	
≥ 151	5.033 (1.050-24.138)	0.043
Acute kidney injury		
No	1.000	
Yes	5.071 (1.235–20.825)	0.024
Nasogastric tubing		
No	1.000	
Yes	5.505 (1.316–23.035)	0.020

### Analysis of pathogenic bacteria in VAP patients after cardiac surgery

After analysis, 31 of the 61 VAP patients tested positive for the pathogenic bacterium, and 19 of the 31 had more than 2 pathogenic bacteria cultivated in their secretions. Meanwhile, no pathogenic bacterium was detected in the specimens of the other 30 patients whose clinical features and imaging performance were consistent with the diagnostic criteria of VAP. A total of 60 pathogenic strains were isolated from the 61 patients, predominantly Gram-negative bacteria (n = 54, 90.0%), including *A. baumannii* (n = 24, 40.0%) and *Klebsiella pneumoniae* (*K. pneumoniae*) (n = 10, 16.7%). However, the detection rate was lower when the Gram-positive bacteria were *Streptococcus pneumoniae* (n = 1, 1.7%). In addition, a total of 5 strains of *Candida* were detected (Table 4). Then, we tested the resistance of Gram-negative bacteria to antimicrobial drugs. *A. baumannii* showed a high rate of resistance but was less resistant to Tigecycline, which may therefore have better therapeutic efficacy against *A. baumannii*. In contrast, *K. pneumoniae* was less resistant to each antimicrobial drug and could be easier to treat.

**Table 4:** Constituent ratios of pathogens isolated in patients with VAP

Pathogenic bacterium	Number of strains [n (%)^a^]	MDRO [n (%)^b^]	Non-MDRO [n (%)^c^]
**Total number**	60(100)	33(55.0)	27(45.0)
**Gram-negative bacteria**	54(90.0)	32(59.3)	22(40.7)
Acinetobacter baumannii	24 (40.0)	23(95.8)	1(4.2)
Klebsiella pneumoniae	10 (16.7)	3(30.0)	7(70.0)
Enterobacter cloacae **Others**	5(8.3) 15(25.2)	1(20.0) 5(333.3)	4(80.0) 10(666.7)
**Gram-positive bacteria**	1(1.7)	1(100)	0(0.0)
Streptococcus pneumoniae	1(1.7)	1(100)	0(0.0)
**Fungi**	5(8.3)	0(0.0)	5(100)
Candida albicans	2(3.3)	0(0.0)	2(100)
Candida tropicalis	3(5.0)	0(0.0)	3(100)

a: Number of strains detected and percentage of the total number of strains; b: Number of MDR strains detected and the percentage of the strains; c: Number of non-MDR strains detected and the percentage of the strains

### Prognosis of patients with VAP after cardiac surgery

Finally, we analyzed the factors affecting the prognosis of patients with VAP after cardiac surgery. Results showed that MDRO VAP patients spent longer postoperatively in the ICU and had prominently higher hospitalization costs than non-MDRO VAP patients. In general, VAP patients had a higher overall mortality rate within 28 days after surgery, but there was no significant difference between the two groups (Table 5).

**Table 5:** Outcome analysis for the patient with VAP [n (%)]

Outcome	Total number (n = 61)	Non-MDRO VAP (n = 34)	MDRO VAP (n = 27)	***P***
Number of days in hospital after surgery	15 (9.5,25.5)	14 (7.8,23.5)	17 (13,27)	0.082
Number of days in ICU after surgery	13 (8.5,17)	9.5 (7.0,15.3)	15 (11,23)	0.007
Death within 28 days after surgery				0.640
No	20 (32.8)	12 (35.3)	8 (29.6)	
Yes	41 (67.2)	22 (64.7)	19 (70.4)	
Total hospitalization cost (million RMB)	33.3 (23.7,43.6)	28.1 (21.9,37.7)	38.7 (29.6,46.2)	0.009

## Discussion

With increasing MDRO, sepsis may lead to high morbidity and mortality, and VAP is one of the most common fatal sepsis in hospitalized patients [11]. Therefore, it is imperative to carefully evaluate the risk of acquiring MDRO pathogens and the clinical severity to find a targeted and rapid diagnosis method. Analyzing the risk factors of VAP caused by MDRO is an effective method.

AKI is a cause of high mortality in septic patients, with an incidence of up to 60% in the ICU [12, 13]. In critically ill trauma patients, AKI is closely linked to VAP [14]. Eduardo et al. indicated that chronic renal failure, cardiopulmonary bypass time, and AKI are independent risk factors for in-hospital death in patients undergoing extracardiac surgery [15]. In line with this, multivariate analysis in our study showed that preoperative creatinine clearance ≥ 86.6, postoperative acute kidney injury, and nasogastric tubing were risk factors for MDRO VAP infection. A previous study noted that enteral nutrition given via the nasal-enteral route was effective in reducing the incidence of VAP and improving the nutritional status of mechanically ventilated patients [16].

Moreover, our study confirmed that an extracorporeal circulation time ≥ 151 min was a risk factor for MDRO VAP infection. The study by He and colleagues was consistent with this view. Also, the duration of intraoperative extracorporeal circulation has been validated as a risk factor for postoperative VAP infection in patients undergoing cardiac surgery [8]. Although not retained in the multifactorial model, the risk factor for MDRO VAP infection in the univariate model was aortic block duration ≥ 77 min. In addition, we found that a preoperative hospital stay of less than 3 days was an independent risk factor for MDRO VAP. Joseph et al. suggested that a longer hospitalization time before VAP diagnosis is a risk factor for MDRO VAP infection [17]. Nasreen and colleagues revealed that postoperative VAP in patients undergoing cardiac surgery is associated with higher mortality and longer hospital stays [7]. However, this is different from our findings. We analyzed that patients with shorter hospital stays are more likely to be admitted to the emergency department, especially those requiring emergency surgery such as aortic coarctation, and are more vulnerable to infection [18].

The most common pathogen causing VAP was *Enterobacteriaceae*, which accounted for 32.8% of the total VAP, followed by *Pseudomonas aeruginosa* (28.6%) and *Staphylococcus aureus* (27.1%) [19]. In detecting MDRO pathogens in VAP patients, we found that *Gram-negative bacteria* were the predominant group of infections, including *K. pneumoniae* and *A. baumannii* that had a high rate of resistance to Imipenem, Meropenem, Ciprofloxacin, Ceftazidime, Ceftriaxone Sodium, Cefepime, Ampicillin/Sulbactam, and Tetracycline. Although the bacteria distribution was different from that in foreign studies, it was consistent with some domestic research [20–22]. It should be noted that the bacteria distribution varies not only from country to country but also from region to region. Thus, as things stand, *A. baumannii* infection has become an emerging health problem in hospitals worldwide [23]. VAP caused by *A. baumannii* can lead to high mortality and healthcare costs, which are dramatically associated with its wide-ranging antibiotic resistance [24, 25]. This is consistent with our results. An earlier study illuminated that colistin is currently the most effective antibiotic for treating *A. baumannii* infection [26]. In contrast, we found that *K. pneumoniae* was less resistant to both colistin and meropenem. And a previous study showed that the colistin-meropenem combination was superior to colistin monotherapy in the treatment of MDR *K. pneumoniae*-induced VAP [27].

Finally, we analyzed the prognosis of VAP patients. The results showed that patients in the MDRO VAP group had a higher mortality rate at 28 days after surgery compared to the non-MDRO VAP group, but there was no significant difference between the two groups. These findings concurred with previous studies. Feng et al. analyzed 106 VAP cases and observed that the 30-day mortality rate in VAP patients was 42.5% [21]. Chand and colleagues indicated that MDRO infection resulted in higher mortality and morbidity in newborns [28]. Nonetheless, there are some limitations that should be addressed. Firstly, the present study included a small size of population which may lead to different conclusions from other studies. Secondly, the retrospective nature of this study requires more samples and multicentered and prospective studies for further verification. It is also important to note that the cost of VAP treatment was greatly increased [29]. Our results revealed significant differences in the length of postoperative ICU stay and the total cost of hospitalization between the two groups, indicating that VAP caused by MDRO not only poses a great challenge to clinical treatment but also brings more risks and difficulties to patients and their families. Therefore, based on the strict implementation of measures related to VAP prevention, more attention should be paid to the patients with risk factors associated with MDRO VAP infection.

In conclusion, our study demonstrates that more importance needs to be attached to adult patients with renal disease, longer intraoperative extracorporeal circulation, and postoperative nasogastric tubing as these features may predispose them to VAP caused by MDRO after cardiac surgery. To develop a better and accurate model for detecting risk factors for MDRO VAP, we will expand the study cohort with further validation and findings.

## Data Availability

The authors agree on sharing this study data and its deposit in public repositories, upon reasonable request.
